# Addressing the need for an operational research framework for Africa

**DOI:** 10.4102/jphia.v17i1.1491

**Published:** 2026-02-06

**Authors:** Isaac A. Choge, Neema W. Kamara, Merawi T. Aragaw, Nicaise Ndembi, Landry D. Tsague

**Affiliations:** 1 Department of Emergency Preparedness and Response (EPR), Africa Centres for Disease Control and Prevention, Addis Ababa, Ethiopia; 2 Department of Surveillance and Disease Intelligence, Africa Centres for Disease Control and Prevention, Addis Ababa, Ethiopia; 3 International Vaccine Institute, Kigali, Rwanda; 4 Department of Primary Health Care, Africa Centres for Disease Control and Prevention, Addis Ababa, Ethiopia

**Keywords:** Africa CDC, emergencies, operational research, implementation research, diseases threats, prevention

## Abstract

Africa Centres for Disease Control and Prevention (CDC), which supports all 55 member states (MS) in Africa, was established to strengthen the capacity and capability of Africa’s public health institutions to detect and respond quickly and effectively to disease threats and outbreaks based on science, policy, and data-driven interventions and programmes. This function is drawn from the African Union (AU) Assembly of Heads of State and Government, which adopted the statute of the Africa CDC in January 2016 and implemented it in mid-2017. Africa desperately needs an operational research (OR) framework for all 55 MS. The OR framework’s strategic aim is to strengthen the capacity to initiate, design and implement OR during disease outbreaks and emergencies. The framework, with support from the Africa CDC, will therefore provide a concise guide to institutional structures, procedures and standards, as well as an outline of methodologies and tools for conducting OR that is context-specific, while promoting the use of generated evidence for policy and decision makers. Drawing on expert reviews, we present here the need for the OR framework and the process of its development for research during public health emergencies in Africa.

## Introduction

Globally, the scale and impact of both disease and natural hazards continue to threaten those most at risk and reverse milestones gained in ensuring public health for all. According to the WHO Global Public Health Intelligence Report estimates, between 2004 and 2023, a total of 5910 health-related events were reported, averaging 296 events per year, and 25% (approximately 74) of these occurred in the African Region.^1^ Africa Centres for Disease Control and Prevention (CDC) estimates that between 2020 and 2023, approximately 5% of the reported events were classified as environmental (related to climate change and fragile states based in conflict zones), 15% related to animal health outbreaks, and 69% related to human health (often in the context of chronic underdevelopment and most at risk of natural, technological and health disasters).^2^ Every year, these crises account for over 227 million years of healthy life lost and an annual productivity loss of over $800 billion.^3^ Left unchecked, the cumulative effect of these public health disasters negatively impacts the socio-developmental course of Africans, reverses the gains of sustainable development goals (SDGs)^4,5^ often requires expensive remedial action, and will have disastrous effects on individuals and communities.

Despite its early developments, implementation research, sometimes interchangeable with operational research (OR), has become a mainstay in virtually all health implementation frameworks.^6,7^ With its roots in strengthening health delivery in clinical settings,^8,9,10^ OR in general has been defined as research that provides for the optimisation of service delivery. By extension, OR identifies gaps in services that are sub-optimal, leading to missed populations and opportunities for greater health, social and economic optimisation. This definition assumes that the research carried out during programmes in particular addresses the shortcomings. Global frameworks on OR exploit the practical approaches to improve implementation and to enhance equity, efficiency, scale-up, and sustainability, and ultimately to improve people’s health.^11^ According to Hales et al., by extension, OR uses an existing resource – the data routinely collected by programmes – to provide ways of improving programme operations and thereby delivering more effective, efficient and equitable care.^12^ Key frameworks from the World Health Organization (WHO) on OR include implementation research in health.^13,14,15^

A key anchor of this strategy is the African Union Scientific, Technical and Research Commission (AU-STRC), which is the main agency responsible for research within the AU. Established in 1965, AU-STRC has been a key player in promoting science and technology across the continent for the past five decades. The AU-STRC is a specialised institution of the AU, and its work is closely linked with the African Union Commission’s broader efforts to promote research and innovation. Following the adoption of the Statute of the Africa CDC and its establishment in 2017, the Africa Health Strategy (AHS 2016–2030) was developed to provide strategic guidance for the implementation of health policies and stipulates the role that the Africa CDC will undertake in disease prevention, surveillance and emergency preparedness and response.^16^ It is within this context that a gap has emerged, leading to the absence of harmonised health information exchange guidelines and standards across member states (MS). These challenges impede comprehensive emergency response-based health research on the continent. Indeed, limited integration of human, animal and environmental health data into surveillance data and infrequent sharing of the (surveillance) data and OR between MS significantly restricts accurate risk mapping and preparedness efforts, especially for the Africa CDC. However, this gap can be bridged by an OR framework on emergency public health response. This will ensure evidence-informed decision-making in the African context before, during and after an event(s) is utilised for purposes of learning, better future response, and building preventative measures. To our knowledge, OR, especially in emergency response settings, is limited, if any, and certainly there is no Africa-wide framework(s) that addresses research priorities during epidemic outbreaks. This therefore remains a key cornerstone for Africa through Africa CDC strategic support to MS on how research should be conducted before, during and after public health emergencies and is a call to action to support the building of the OR framework during emergencies for the wider global health community.

## Theory of change

**FIGURE 1:**
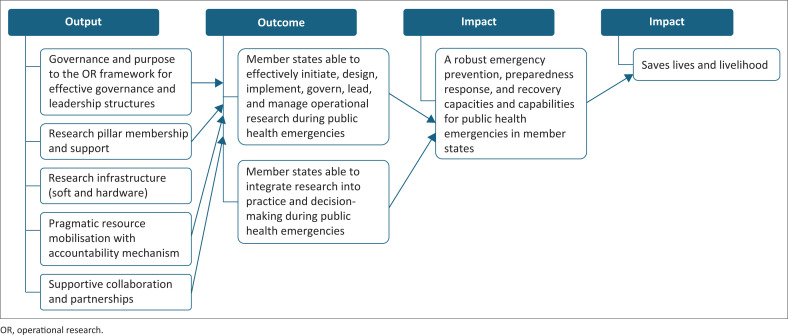
Proposed theory of change for the operational research framework for Africa Centres for Disease Control and Prevention in support of member states during emergencies.

The authors here have limited themselves to an abbreviated theory of change (ToC) as illustrated in Figure 1. The highlights focus on the relatedness of the intended outputs to the impact of the OR framework. Of particular emphasis is the governance and leadership output, which remains the driving force in support of MS.

## Key considerations of the operational research framework for Africa

The final impact of the OR framework will be saving lives through lessons learnt from public health emergency operations. Various characteristics of the development of the OR framework, including workshops with relevant specialists and experts, fieldwork with MS, laboratory studies with support from academic institutions, clinical trials, and the synthesis of existing data and evidence alongside extensive government consultations, have over time been considered key for an OR framework. For Africa CDC, the ensuing guidelines and standards, alongside meta-analyses and commissioned reviews, will be undertaken and will be published in due course. However, for the purposes of this article, we highlight the key areas that were considered in the need for the OR framework for African MS and the Africa CDC. These areas are discussed in further detail.

## Governance and purpose

Embedded within the Africa Health Strategy (AHS 2016–2030), the development of the OR framework was undertaken with the purpose to generate evidence aimed at effective response(s) before, during and after emergency outbreaks, to inform response aspects to public health emergencies including management, inform how future emergencies will be responded to, and document best practices within the African context especially with limited resources and missed opportunities. This includes evidence generated in the respective response pillars, while keeping in mind effective, efficient, sustainable and impactful outcomes as guided by the established terms of reference. The governing areas for the OR framework lie in the leadership with a clear role in providing strategic guidance and direction, ensuring trust, creating influence and building a research culture. In the OR framework, we propose clear linkages with established research governance structures within MS and with coordination mechanisms, including the response incident management system (IMS) in the overall coordination of the response. While keeping in line with MS regulatory frameworks, the respective national regulatory authorities would oversee individual country research activities, including research ethics through national research ethics boards or committees.

## Research pillar membership

At the core of the OR framework is the implementation of research within the emergency response. The function of the emergency research pillar is to coordinate research groups, partners and stakeholders, reporting to the incident manager. Depending on the emergency, this core group would be expanded or contracted from a pool of mapped and trained personnel during emergencies. The characteristics of this research group would remain constant for the duration of the emergency response. An important consideration is the delegated authority from the incident management team to decide and/or propose research priorities and make decisions related to research. Lessons learnt from the Ebola, Marburg and mpox outbreaks remain important cornerstones in how research was conducted during these periods. Alongside decision-making, the core group will be responsible for compiling and validating relevant research questions and overall coordination of all research undertaken during the emergency. The research pillar members will be supplemented with support from various academic and/or research institutions or departments.

## Research infrastructure

A key consideration for the OR framework during emergencies is the infrastructure from four key reference points. Justification of these reference points is drawn by the level of effort during emergencies and the responses undertaken by both Africa CDC and the affected MS. This includes laboratory, information technology, data infrastructure, and equipment and supplies. In short, the laboratory reference points include human and animal testing, and the OR frameworks aim to guide field teams with mobile research units (e.g. mobile labs or clinics) to conduct rapid assessments, testing and sample collection in remote or disaster response areas. The repurposing of laboratories near affected areas aims to reduce sample transport times (and cost) and increases the speed of diagnostic testing or biomedical research.

Information technology directly influences the OR framework as it remains the backbone of any data management. This would ultimately include a reference to the use of cloud-based platforms (e.g. Microsoft Teams, Google Workspace) for collaboration between geographically dispersed research teams. In addressing this gap, this OR framework should therefore align emergency response and the needs of the population through the deployment of telemedicine platforms and remote monitoring technologies for real-time data collection.

Data infrastructure suggests that to effectively have a working framework, data must be collected and used to make decisions in a timely manner.

Equipment and supplies remain the backbone of any research process. The Africa CDC OR framework is envisioned to entrench this reference point given that in Africa there are large demands placed on logistics, equipment, vehicles, consumables (stationery, sampling kits), and more importantly, connectivity such as mobile data collection tools (telephones, toll-free number), communications devices and emergency kits (such as first aid, personal protective equipment, rescue, etc.).

## Resource mobilisation

The need for resources for OR, especially during emergencies, deserves special focus given the already underfunded pillars in healthcare. As the need for an OR framework for Africa becomes imperative, great efforts by the technical experts have been made to guide decision makers in the thought process of the OR framework. The emphasis has been placed on executive orientation on the role of OR in health and economic development. This effort is targeted at unlocking resources through government allocation sources, including commitments of the 15% of gross domestic product (GDP) for health through the Abuja Declaration and implemented by the AU, the need for 5% – 10% of each health programme budget for research and the need for at least 1% of GDP for research and development to be committed by each MS.

## Collaboration and partnerships

The OR framework would need to maintain its agility and dynamic implementation nature, given the wide range of responses it would guide in the differing emergency contexts. This suggests that a continuum of collaborations with local, regional and international partners and stakeholders would be the adhesive to enable successful outcomes. The guidance from the OR would include mapping stakeholders, including government partners, external agencies, private sector collaborators, community support systems, donors, institutions, non-governmental organisations (NGOs), and so on, with whom there is a need to establish action-oriented, respectful partnerships. Likewise, considerations would be given to geographical presence, implications of the research outputs or outcomes, expertise availability and resources available for the research.

To maintain current and future collaborations, purposeful partnerships would be built for better coordination between government ministries, NGOs, academic institutions, open-access journals, regional economic communities and international agencies for resource sharing and synchronised research efforts. These efforts are even more crucial when public health emergencies cross international borders and/or have a multicountry impact.

## Expected outcomes and future directions

The need for an OR framework to guide Africa through the Africa CDC and the AU MS during public health emergencies is overdue. The gap presented by the lack of the OR framework suggests that current research is both haphazard, inefficient, ineffective and will have less impact on the lessons learnt through continuity for the future and responding to emergencies in the African context. With over 180 emergencies every year, this OR framework will shape how Africa documents its lessons learnt, where resources can be attained and used, which partners to work with, and most importantly, how to save lives. As of 2021, 20 years after agreeing to the Abuja Declaration and committing to spend at least 15% of their national budgets on healthcare, only two of the AU’s 55 MS met this target in 2021: Cabo Verde (15.75%) and South Africa (15.29%). Alongside the same timeframe, AU MS spent an average of 7.35% of their national budgets on healthcare, including research.^16^ At the same time, the emergency response expenses have markedly increased. These low expenditure patterns in programming of healthcare and high expenditures on emergency responses suggest that Africa can use this opportunity to fully develop the OR framework to learn from its emergency operations before, during and after responses to public health incidents. That would mean responses that are more efficient, effective, sustainable and have an impact on the population affected in terms of saving lives. It is in this framework that Africa CDC can step in and guide how efforts in otherwise expensive emergency responses can be attained. In turn, AU MS can better utilise their efforts to better plan for emergency responses. It is this argument presented here that the need for an OR framework should not be further delayed, but supported fully for its full implementation. Finally, we present in this article a simplified ToC, illustrating the need for and some of the thoughts behind the OR framework, what should be included and who will most likely benefit. Notwithstanding the gaps identified by the absence of a guiding OR framework for MS, one major challenge of such proposed initiatives is the funding mechanism for execution. In-depth analyses have been summarised by the works of Ndembi et al.^17^ not only in financing public health prevention, preparedness and response, but also in what future outlooks and options are available. However, the key to financing research activities remains unanswered for continental bodies like the Africa CDC. This article lays the foundation for the completion of the OR framework and through this call, we have attempted to chart the future direction in an otherwise void OR framework setting.
